# Change in Splenic Volume as a Surrogate Marker for Immunotherapy Response in Patients with Advanced Urothelial and Renal Cell Carcinoma—Evaluation of a Novel Approach of Fully Automated Artificial Intelligence Based Splenic Segmentation

**DOI:** 10.3390/biomedicines11092482

**Published:** 2023-09-07

**Authors:** Gregor Duwe, Lukas Müller, Christian Ruckes, Nikita Dhruva Fischer, Lisa Johanna Frey, Jan Hendrik Börner, Niklas Rölz, Maximilian Haack, Peter Sparwasser, Tobias Jorg, Christopher C. M. Neumann, Igor Tsaur, Thomas Höfner, Axel Haferkamp, Felix Hahn, Rene Mager, Maximilian Peter Brandt

**Affiliations:** 1 Department of Urology and Pediatric Urology, University Medical Center, Johannes-Gutenberg University Mainz, Langenbeckstrasse 1, 55131 Mainz, Germany; 2 Department of Diagnostic and Interventional Radiology, University Medical Center, Johannes-Gutenberg University Mainz, Langenbeckstrasse 1, 55131 Mainz, Germany; 3 Interdisciplinary Center for Clinical Trials Mainz, University Medical Center, Johannes Gutenberg-University Mainz, Langenbeckstrasse 1, 55131 Mainz, Germany; 4 Department of Hematology, Oncology and Tumor Immunology, Charité-Universitätsmedizin Berlin, Freie Universität Berlin, Humboldt-Universität zu Berlin, Berlin Institute of Health, 10117 Berlin, Germany; 5 Department of Urology, Ordensklinikum Linz Elisabethinen, Fadingerstraße 1, 4020 Linz, Austria

**Keywords:** advanced urothelial carcinoma, advanced renal cell carcinoma, immunotherapy, immune checkpoint inhibitor, prognostic marker, predictive marker, splenic volume, artificial intelligence

## Abstract

Background: In the treatment of advanced urothelial (aUC) and renal cell carcinoma (aRCC), biomarkers such as PD-1 and PD-L1 are not robust prognostic markers for immunotherapy (IO) response. Previously, a significant association between IO and a change in splenic volume (SV) was described for several tumour entities. To the best of our knowledge, this study presents the first correlation of SV to IO in aUC and aRCC. Methods: All patients with aUC (05/2017–10/2021) and aRCC (01/2012–05/2022) treated with IO at our academic centre were included. SV was measured at baseline, 3 and 9 months after initiation of IO using an in-house developed convolutional neural network-based spleen segmentation method. Uni- and multivariate Cox regression models for overall survival (OS) and progression-free survival (PFS) were used. Results: In total, 35 patients with aUC and 30 patients with aRCC were included in the analysis. Lower SV at the three-month follow-up was significantly associated with improved OS in the aRCC group. Conclusions: We describe a new, innovative artificial intelligence-based approach of a radiological surrogate marker for IO response in aUC and aRCC which presents a promising new predictive imaging marker. The data presented implicate improved OS with lower follow-up SV in patients with aRCC.

## 1. Introduction

Over the last decade, immunotherapy (IO) has become the standard of care in the treatment of advanced urothelial carcinoma (aUC) and advanced renal cell carcinoma (aRCC) [1,2]. Despite a heterogeneous patient population, these patients are severely ill with a relatively moderate-to-poor prognosis with the 5-year survival chance rated less than 15% for patients with aUC. Therefore, a continuous evaluation of therapy response in the context of patient-centred therapy is of great importance. This is particularly true for patients who undergo surgical resection and receive chemotherapy or other therapies such as immunotherapy. Until today, the PD-1, as well as the PD-L1 expression, has not proven to be a robust biomarker for therapy response in aUC and aRCC. Besides multiple prognostic models, no relevant radiological surrogate markers exist to detect early IO response in these patients [3,4]. 

In aUC, two monoclonal antibodies targeting programmed cell death protein (PD)-1 (nivolumab and pembrolizumab) and three antibodies targeting PD-ligand (L) 1 (atezolizumab, avelumab, and durvalumab) were approved by the U.S. Food and Drug Administration (FDA) to treat aUC [5,6]. Since 2016, IO agents have become firmly established in nearly all stages of aUC. IO has become particularly important for elderly/fragile patients who cannot always be treated by platinum-based regimens (i.e., cisplatin or carboplatin) [7]. Nevertheless, complete response rates of modern IO approaches in various treatment settings are only around 10% and the objective response rates range from 10 to 55%. Therefore, valid prognostic factors are crucial to avoid unnecessary, expensive therapies with multiple potential adverse events and thus enable real patient-individualized, targeted therapies [8]. IO has a long history in the systemic treatment of aRCC because RCC is not responsive to chemotherapy at all [2]. Notably, approximately 15% of patients with RCC present with aRCC. Besides PD-1 and PD-L1 inhibitors, other targeted antibodies and various treatment combinations, e.g., anti-cytotoxic T-lymphocyte-associated protein 4 (CTLA-4) antibodies (ipilimumab), vascular endothelial growth factor (VEGF) inhibitors (axitinib, sunitinib, pazopanib, cabozantinib, lenvatinib, and bevacizumab), or mammalian (mechanistic) target of rapamycin (mTOR) inhibitors (everolimus) exist [9]. Despite a longer experience with the use of IO in aRCC compared to aUC, there are still „no effective markers to select patients who might benefit from immunotherapy and to guide therapeutic strategies“ [3]. Currently published data from 2022 using multiple tumour microenvironments and biomarkers with up to 5-gene panels in a cohort of aRCC patients receiving various IOs, did not achieve predictive values for radiologic response, progression-free survival (PFS), or (OS) [10]. 

Over the last few years, a change in splenic volume has been investigated as new surrogate marker for patients with several malignant diseases receiving IO. This approach is of great interest due to the easily available assessment of SV during routine imaging in the context of the underlying disease. The pathophysiological background for these prior and current investigations to date is based on studies which assessed the spleen as a lymphoid organ under treatment with IO. Two in vivo models reported that the anti-programmed death-ligand 1 (PD-L1) treatment increased the percent population of monocytes/macrophages, CD8+ cells and natural killer cells in the spleen [11,12]. Furthermore, an animal-experimental study showed that high-dose ipilimumab plus nivolumab led to an increased proliferation of CD4+ and CD8+ lymphocytes as well as an increase in activated T cells and central memory T lymphocytes in the spleen, which was accompanied by an enlargement of the spleen [13]. Based on this scientific evidence, a hypothesis was designed that changes in SV during therapy with IO may be related to changes in the number and function of immune cells in the spleen; thus, the changes in SV could serve as a surrogate marker for IO treatment response [14,15]. 

A novel and promising method using a simple automated radiological tool was described by Müller L et al. in 2022. They used a previously developed fully automated artificial intelligence (AI)-based splenic segmentation software (Version 1) [16] to predict survival outcomes in patients with hepatocellular carcinoma under IO [17]. Moreover, SV increase was also described by other research teams in melanoma patients who received immune checkpoint inhibitors [14] as well as in patients with metastatic non-small cell lung cancer [15,18]. Thus, we regard this AI-based method as a promising imaging biomarker with the potential for full integration into the routine radiology workflow.

In summary, there is a rapidly changing therapeutic landscape in the treatment of aUC and aRCC that is leading to significant improvements in patient outcomes [8]. However, there is still an urgent need for simple and feasible prognostic tools to detect early responses to IO treatments [3,4]. Since IO as described has taken an important place in the treatment of aUC and aRCC in recent years, we aimed to investigate both tumour identities separately in our study in order to make a new, innovative, scientific contribution to the treatment of genitourinary cancer. However, it is important that both outcomes are assessed separately because patients with aUC have, on average, a worse prognosis compared to patients with aRCC. Moreover, patients with aUC are subjected to IO approaches more often in subsequent treatment lines following chemotherapy, and therefore have less chance of treatment efficacy and overall survival, compared to patients with aRCCs which instead are frequently subjected to IO in the first lines.

To the best of our knowledge, this is the first study examining the role of SV and changes in SV in patients with aUC and aRCC who have received IO. Thus, we aimed to assess this novel approach of AI-based fully automated assessment of the SV using computed tomography (CT) data to evaluate initial change in SV as a new imaging biomarker for patients with aUC and aRCC treated with IO.

## 2. Materials and Methods

### 2.1. Patients

**Figure 1:**
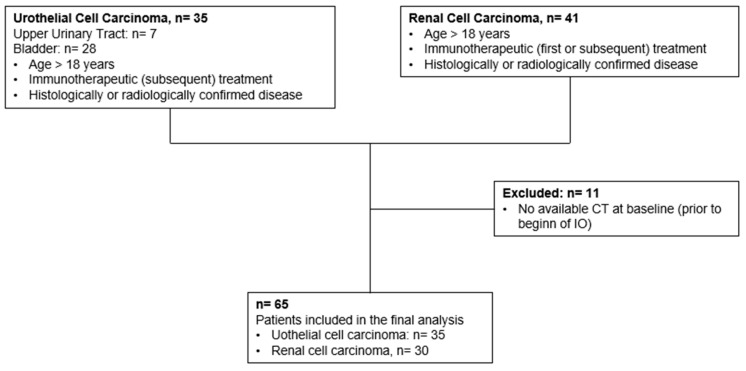
Flowchart of the patient selection process of this study.

This retrospective study included all patients who presented in our in- and outpatient clinic between May 2017 and October 2021 for aUC and all patients with aRCC between January 2012 and May 2022 for the initiation or subsequent treatment of IO. The requirement for informed consent was waived due to the retrospective nature of the study. This report followed the guidelines for reporting observational studies (STROBE) [19]. Inclusion criteria were age > 18 years, histologically derived UC or RCC diagnosis, IO as systemic treatment, CT images available prior to IO, as well as at least three and nine months after therapy initiation, and demographic, clinical, and pathological data available at initiation of the IO. Of the scanned 76 patients, 65 (85.53%) patients fulfilled all inclusion criteria (Figure 1). The decision to initiate immunotherapy was made by an interdisciplinary tumour board. If not available, all patients received contrast-enhanced multiphasic CT imaging of the chest and abdomen or a magnetic resonance imaging of the abdomen (MRI) prior to treatment initiation. The follow-up consisted of clinical examination, blood sampling, and cross-sectional imaging.

### 2.2. Splenic Volume Assessment

SV was assessed using an established tool for fully automated segmentation and volumetry of the spleen as described previously [16]. This algorithm employs the open-source MIScnn library, a convolutional neural network with a U-Net architecture, and has previously been trained for spleen segmentation in patients with HCC undergoing transarterial chemoembolization (TACE) [20] as well as patients with HCC under IO [17]. Further detailed information on the features of the network, the settings for training and validation, and the model’s performance can be found in the original publication [16]. A radiologist with 4 years experience in abdominal imaging checked all created segmentations separately. In our dataset, the quality of the graphic overlays was poor in only one case. Thus, for this case manual segmentation with the freely available LIFEx software (Version 7.3.0) was performed (www.lifexsoft.org, accessed on 1 October 2022) [21]. In the second step, SV was normalized to the body surface area (BSA), which was calculated using the patient’s height and weight.

### 2.3. Statistical Analysis

The statistical analysis was performed using IBM SPSS Statistics Version 27 (Armonk, NY, USA: IBM Corp.) and SAS software Version 9.4 (SAS Institute Inc., Cary, NC, USA). Continuous variables are presented as medians ± interquartile range (IQR) in accordance with the data contribution. Categorical and binary baseline parameters were reported as absolute numbers and percentages. For all patients, OS and progression-free survival (PFS) under IO treatment were calculated from the initiation of treatment. Log-rank testing was used to compare survival times. Cox proportional hazards regression models assessing hazard ratios (HRs) and corresponding 95% confidence intervals (CIs) were used to determine the effect of the risk stratification. All tests were 2-tailed with *p* < 0.05 and considered statistically significant. 

## 3. Results

### 3.1. Baseline Characteristics of Patients with Advanced Urothelial Carcinoma

Baseline characteristics of the study population with aUC are provided in Table 1. A total of 35 patients, 24 males (68.6%) and 11 females (31.4%), with a median age at the beginning of IO of 65 years (interquartile range: IQR: 57.50; 73.50 years), were included in the final analysis. Baseline and follow-up CT scans were available for all patients. In total, 30 patients (85.7%) received adjuvant or palliative chemotherapy, the median time from initial diagnosis until recurrence was 11.00 months (IQR: 5.0; 31.00), and the follow-up was 11.50 months (IQR: 6.25; 24.25). At the last follow-up (15 June 2023), 18 patients (51.4%) had died.

### 3.2. Baseline Characteristics of Patients with Advanced Renal Cell Carcinoma

Baseline characteristics of the study population with aRCC are provided in Table 2. A total of 30 patients, 26 males (86.7%) and 4 females (13.3%), with a median age at the beginning of IO of 66 years (IQR: 59.00; 72.00 years), were included in the final analysis. Baseline and follow-up CT scans were not available for 11 patients as they had to be excluded (see Figure 1). At the time of the initial diagnosis, 8 patients (26.7%) presented with positive regional lymph nodes, while 11 patients (36.6%) already had distant metastasis. Until now, the overall follow-up was 25 months (IQR: 13.00; 40.00) with 10 patients (33.3%) deceased.

### 3.3. Change in Splenic Volume after Initiation of Immunotherapy

The median baseline SV for all patients was 271.80 mL (IQR: 170.65; 296.54 mL). At the three-month follow-up CT scan after beginning IO, the median SV was 252.93 mL (IQR: 175.00; 315.30), and at the 9-month follow-up, it was 244.93 mL (IQR: 191.25; 279.48). The changes in SV by tumour identity are shown in Table 1 and Table 2. An increase in the SV at the three-month follow-up was observed in 31 (47.7%) patients, whereas 33 (50.8%) patients had a decrease in SV during early treatment (missing data for one patient). The median change in SV at three-month follow-up was 1.34 mL (IQR −15.80; 35.03 mL, range −98.57–78.14 mL). Next, an increase in the SV at the nine-month follow-up (compared to baseline SV) was observed in 15 (23.1%) patients, whereas 18 (27.7%) patients had a decrease in SV during early treatment (missing data for 32 patients). The median change in SV at the nine-month follow-up was −9.17 mL (IQR −35.67; 25.67 mL, range −94.73–105.36 mL). 

### 3.4. Impact of Splenic Volume at Treatment Initiation and during Three-Month Follow-Up on Overall Survival

**Figure 2:**
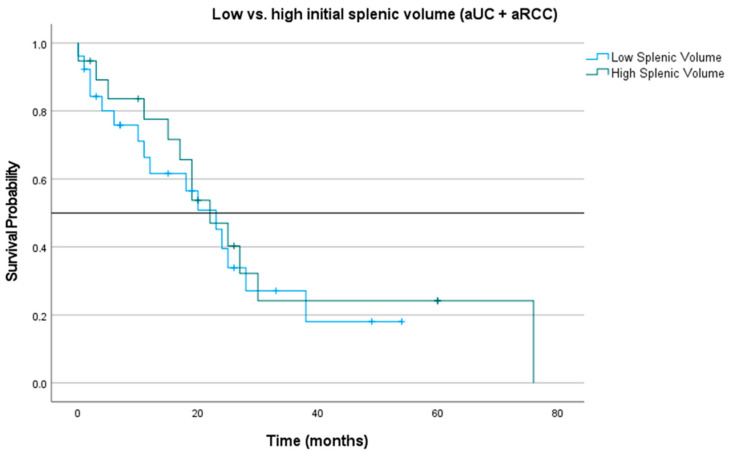
Kaplan–Meier curve for patients (aUC and aRCC) with initial low and high splenic volume. Blue line representing patients with initial low splenic volume, green line representing patients with initial high splenic volume.

First, patients were dichotomized into low and high SV based on the median SV of the total patient cohort. The median (OS) of patients with low SV at baseline was 23.00 months (CI 15.33; 30.68), whereas patients with initial high SV had a median OS of 22.00 months (CI 14.41; 29.59; *p* = 0.188), see Figure 2. 

**Figure 3:**
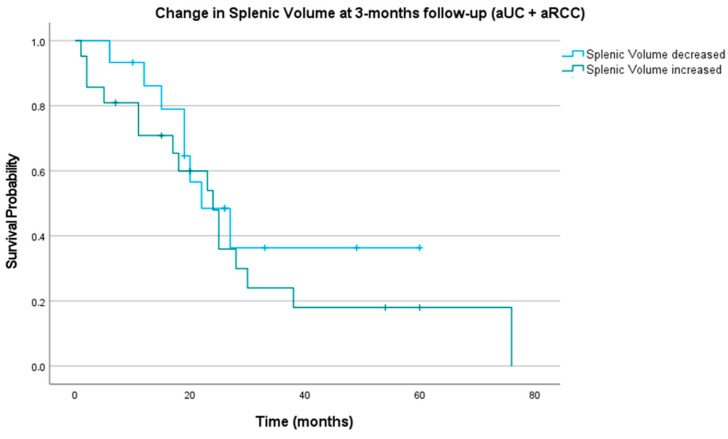
Kaplan–Meier curve for patients (aUC and aRCC) with decrease and increase in splenic volume three months after start of IO. Blue line representing patients with decrease in splenic volume, green line representing patients with increase in splenic volume.

Subsequently, we investigated the survival of patients with a decrease and increase in SV at the three-month follow-up. Patients with a decrease in SV at the three-month follow-up had a median OS of 22.00 months (CI 12.65; 31.35), whereas patients with an increase in SV at the three-month follow-up had a median OS of 24.00 months (CI 17.33; 30.68; *p* = 0.643), see Figure 3.

### 3.5. Multivariate Survival Analysis for Overall Survival and Progression-Free Survival Based on Initial Splenic Volume and Change in 3Month Follow Up Splenic Volume

Next, we evaluated several risk factors on OS and PFS by multivariable cox proportional hazards model. For patients with aUC we determined no clinical variables to significantly influence OS as well as PFS, presented in Table 3. For patients with aRCC we stratified patients with initial low SV (dichotomized into low and high SV based on the median) as well as with low SV at the three-month follow-up, see Table 4. In this analysis, there was a significant positive correlation between a low splenic volume at the three-month follow-up and OS (HR: 0.007, CI: 0.01–0.91, *p*-value: 0.041). All other comparisons did not reveal statistically significant results.

## 4. Discussion 

In this study, we investigated the role of SV and changes in SV with regard to survival and progression-free survival as a marker of therapy response after initiation of immunotherapy in patients with aUC and aRCC. This approach offers an interesting and feasible clinical methodology to evaluate and predict the treatment response for patients under IO. On the one hand, cross-sectional imaging with an abdominal CT is available in almost all patients with aUC and aRCC under treatment with IO and, on the other hand, the evaluation can be performed in an AI-based automated manner. Interestingly, despite non-significant results in the aUC group, as well as baseline SV volume differences in the aRCC group, a low SV at the three-month follow-up in the aRCC group was significantly associated with an improved OS in the multivariate analysis. 

The essential background of the present study lies in the lack of prognostic tools for patients with (genitourinary) cancer receiving IO. Based on multiple large, randomized phase III trials that lead to the approval of various IOs in aUC and aRCC, we know that not all patients benefit equally from IO in terms of OS and PFS. In clinical practice, progression or tumour recurrence is commonly based on radiological follow-up images in order to adapt the therapy if necessary. Therefore, also due to the high costs of IO, a good, feasible surrogate marker for response to IO is of great interest.

In 2021, Rebuzzi et al. reviewed the current prognostic and predictive factors for treatment with immune checkpoint inhibitors in aUC [4]. They summarized various variables (molecular classes, tumour mutational burden, mutational signatures, cell-free DNA (ctDNA), PD-L1, patient’s characteristics, concomitant medications, inflammatory indices, combined tools, and radiomic-based modes) and evaluated their strength of evidence and clinically meaningfulness. In short, only ctDNA [22,23] and clinical factors summarized by Bellmunt et al. and Bajorin et al. [24,25] were valued as clinically meaningful prognostic parameters while only ctDNA was additionally evaluated as a clinically meaningful predictive parameter [4]. They also reviewed major studies addressing the prognostic and predictive value of radiomics, which resemble the quantitative analysis of imaging features by artificial intelligence (AI) algorithms. However, the overall results are not yet sufficient for routine clinical use and need further validation [26,27]. In the clinical routine, the Bellmunt Risk Score, first described in 2010 and based on Eastern Cooperative Oncology Group (ECOG) performance status (PS), haemoglobin levels, and the presence of liver metastases, is the most commonly applied prognostic tool which does, however, only apply to patients with aUC progressing after platinum-based chemotherapy [25]. Against this background, Bamias A et al., Merseburger A, and Loriot Y et al. presented a new prognostic model in January 2023 for patients with aUC receiving atezolizumab after platinum-based chemotherapy (phase IIIb SAUL trial) [28]. They suggested that the three-factor Bellmunt risk score can be significantly improved for patients receiving second-line IO by adding alkaline phosphatase, neutrophil-to-lymphocyte ratio, bone metastases and time from last chemotherapy in a four-tier model. The problem is much more serious in patients with aRCC, where there are still no established prognostic markers for IO response at all or promising studies of at least potential makers published so far [3].

To date, there are only very few studies that use SV for evaluation as a surrogate marker of IO efficacy. Susok et al. published a small cohort of 49 stage III and IV melanoma patients and reported an increase in SV after 3 months of follow-up, particularly with the use of anti-CTLA-4 and anti-CTLA-4/anti-PD-1 regimens [14]. However, they did not report any further data of their statistical analysis as well as no further significant relationship with other clinical parameters. In 2021, Galland et al. reported more detailed and promising data on 276 patients with non-small lung cancer, representing the largest retrospective study cohort in this comparison of published data in this research field [18]. They were able to demonstrate a statistically significant correlation of initial SV and change in SV (increase in SV in 64.5% of patients being associated with impaired OS) with OS. A similar study, but with negative results, was published in 2022 by Castagnoli et al. [15]. They investigated the change in SV in 70 patients with non-small cell lung cancer who received pembrolizumab and reported no significant differences in patients showing clinical benefit versus those without clinical benefit to pembrolizumab. These examples for non-small lung cancer illustrate the need for further evaluation using larger patient cohorts to verify the contradictions of those initial results.

In 2022 Müller et al. investigated for the first time the change in SV in a cohort of 55 patients with HCC receiving IO [17]. They demonstrated a significant correlation of high baseline splenic volume (SV) with impaired OS (4.0 months vs. 30.7 months, *p* = 0.004). Additionally, they achieved these results based on fully automated AI-based SV assessment. Manuel spleen segmentation is time-consuming and has a high risk of inter-rater variance [29]. Thus, AI-based automated SV assessment has a clinically highly relevant potential to facilitate and standardize this task, thus enabling easy integration into a radiological routine. The feasibility of this technique has already been demonstrated [16,30]. The present study confirms the easy integration of SV assessment into the routine workflow, together with the first time-reported prognostic importance of SV for patients with aRCC undergoing IO. 

There were several limitations in our study similar to the previous studies reported. First, this study was conducted in a retrospective manner and included a limited number of patients. The low number of patients might be explained by the monocentric design of this study in an area that does not have a dense population. Another reason might be the fact that IO can be given in an outpatient setting; therefore, these patients are not routinely monitored at our tertiary academic centre. Nevertheless, this dataset was well-investigated and only patients with complete clinical and imaging data were included. No imputation of missing values was performed. Thus, based on automated AI-based measurement of SV with feasible clinical application, our study represents pioneering methodological work described for the first time and warrants further validation with larger cohorts, as has been carried out in prior studies with other tumour identities [14,15,17,18]. Second, we decided to include patients with aRCC treated with various immunotherapeutic agents to validate the role of SV in a real-life clinical setting. We did not perform subgroup analysis on each immunotherapy agent due to the small number of patients in each subgroup. However, future studies should validate SV in larger study cohorts as a novel prognostic factor for various immunotherapy agents and treatment lines.

## 5. Conclusions

In conclusion, we report a novel approach of fully automated AI-based SV assessment for a new surrogate marker for the response of IO in patients with aUC and aRCC. To our knowledge, no comparable investigations have yet been published for patients with aUC and aRCC. Interestingly, a lower SV might implicate improved OS in patients with aRCC receiving IO. However, external validation with larger patient cohorts will be needed to evaluate our findings. In addition, we describe a new, innovative technique of a radiological surrogate marker which is easily and inexpensively available in everyday clinical practice.

## Figures and Tables

**Table 1:** Baseline characteristics of patients with advanced urothelial carcinoma.

Characteristics (Numerical)	n = 35	
Age at start of IO		
Median (IQR)	65.00 (57.50; 73.50)	
Time period initial diagnosis until metastasis (months)		
Median (IQR)	11.00 (5.0; 31.00)	
Period from start of IO until progress (days)		
Median (IQR)	143.00 (109.25; 255.25)	
Overall Survival/Follow-Up (months)		
Median (IQR)	11.50 (6.25; 24.25)	
Splenic Volume at baseline (start of IO)		
Median (IQR)	191.84 (152.16; 273.44)	
Splenic Volume at 3-month follow-up		
Median (IQR)	236.51 (181.55; 255.35)	
Splenic Volume at 9-month follow-up		
Median (IQR)	202.74 (135.49; 253;44)	
IQR: interquartile range		
Characteristics (categorical)	n = 35	%
Synchronous Metastasis		
Yes	4	11.4
None	6	17.1
Unknown/data missing	25	71.4
Sex		
Men	24	68.6
Female	11	31.4
Unknown/data missing		
Neoadjuvant * intravesical treatment with BCG		
yes	1	2.9
no	19	54.3
Unknown/data missing	15	42.9
Neoadjuvant * intravesical treatment with Mitomycin C		
yes	6	17.1
no	14	40
Unknown/data missing	15	42.9
Neoadjuvant * chemotherapy		
yes	2	5.7
no	24	68.6
Unknown/data missing	9	25.7
Adjuvant/Palliative chemotherapy		
yes	30	85.7
no	2	5.7
Unknown/data missing	3	8.6
Deceased		
Yes	18	51.4
None/Unknown	17	48.6
IO: immunotherapy		

* prior to cystectomy or nephroureterectomy.

**Table 2:** Baseline characteristics of patients with advanced renal cell carcinoma.

Characteristics (Numerical)	n = 30	
Age at time of initial diagnosis		
Median (IQR)	65.00 (57.00; 72.00)	
Age at start of IO		
Median (IQR)	66.00 (59.00; 72.00)	
Overall Survival/Follow-Up (months)		
Median (IQR)	25.00 (13.00; 40.00)	
Splenic Volume at baseline (start of IO)		
Median (IQR)	281.72 (241.89; 312.85)	
Splenic Volume at 3-month follow-up		
Median (IQR)	298.23 (222.16; 328.43)	
Splenic Volume at 9-month follow-up		
Median (IQR)	260.71 (209.90; 293;12)	
IQR: interquartile range		
Characteristics (categorical)	n = 30	%
Sex		
Men	26	86.7
Female	4	13.3
Histology of RCC		
Clear Cell RCC	24	80
Papillary RCC	2	6.7
Other	4	13.3
Initial local treatment		
Nephrectomy	17	56.7
Partial nephrectomy	3	10
No surgery/other	10	33.3
Regional lymph nodes at the time of initial diagnosis		
Negative (N0)	6	20
Positive (N1)	8	26.7
Not Assessable (Nx)	11	36.7
Unknown/data missing	5	16.7
Distant Metastasis at the time of initial diagnosis		
Negative (M0)	3	10
Positive (M1)	11	36.6
Unknown/data missing	16	53.3
IMDC Score *		
1	13	43.3
2	8	26.6
3	5	16.7
4	2	6.7
Unknown/data missing	2	6.7
Synchronous lymph node metastasis		
yes	10	33.3
no	20	66.7
Synchronous distant metastasis		
yes	11	36.7
no	19	63.3
Distribution of metastases		
Lung	18	60
Liver	3	10
Bones	9	30
Lymph nodes	13	43.3
IO at first-line treatment		
Yes	16	
None/Other	14	
IO at second-line treatment		
Yes	14	
None/Other	16	
IO at third-line treatment		
Yes	7	
None/Other	23	
Deceased		
Yes	10	33.3
None/Unknown	20	66.6
IO: immunotherapy		

* International Metastatic Renal Cell Carcinoma Database Consortium Score.

**Table 3:** Multivariate survival analysis for overall and progression-free survival based on initial splenic volume among patients with aUC treated with immunotherapy.

Patient Characteristic	Cox Proportional Hazards Regression for Survival			
	Overall Survival		Progression-Free Survival	
	HR (95% CI)	*p*-Value	HR (95% CI)	*p*-Value
Initial low SV *	2.21 (0.75–6.51)	0.151	1.99 (0.76–5.20)	0.163
Age at start of IO	0.98 (0.92–1.04)	0.505	0.96 (0.92–1.01)	0.105
Time span from initial diagnosis to recurrence	0.99 (0.99–1.00)	0.095	0.99 (0.99–1.00)	0.208
Time span from recurrence to start of IO	1.00 (0.99–1.00)	0.185	1.00 (0.99–1.00)	0.228

* dichotomized into low and high SV based on the median SV of the patient cohort: low splenic volume = lower 50% by median, HR: hazard ratio, CI: confidence interval, *p*-value: Pr > ChiSq, SV: splenic volume; IO: immunotherapy.

**Table 4:** Multivariate survival analysis for overall survival based on initial and 9-month follow-up splenic volume among patients with aRCC treated with immunotherapy.

Patient Characteristic	Cox Proportional Hazards Regression for Overall Survival			
	Based on Initial Splenic Volume		Based on Follow-Up Splenic Volume	
	HR (95% CI)	*p*-Value	HR (95% CI)	*p*-Value
Initial low SV *	0.25 (0.04–1.78)	0.167		
low SV at 3-month follow-up *			0.07 (0.01–0.91)	0.041
Age at start of IO	0.94 (0.82–1.08)	0.396	0.97 (0.90–1.03)	0.286
T-Stage (pathologically)	0.48 (0.08–2.94)	0.425	0.13 (0.01–1.95)	0.141
N-Stage (pathologically)	0.21 (0.01–3.72)	0.289	3.61 (0.24–54.51)	0.356
IMDC Score	0.36 (0.11–1.34)	0.129		
Charlson Comorbidity Index	2.66 (0.41–17.22)	0.305		

* dichotomized into low and high SV based on the median SV of the patient cohort: low splenic volume = lower 50% by median, HR: hazard ratio, CI: confidence interval, *p*-value: Pr > ChiSq, IO: immunotherapy, IMDC: International Metastatic RCC Database Consortium; T- and N-Stage: <5 versus >5.

## Data Availability

Data cannot be shared publicly because of institutional and national data policy restrictions since the data contain potentially identifying patient information. Data are available upon request for researchers who meet the criteria for access to confidential data.
